# Arkansas Oil-Stone

**Published:** 1846-09

**Authors:** 


*Arkansas Oil-Stone.*—For the information of those who may not be aware of the fact, we take pleasure in stating, that the owners of the quarry of this valuable stone, have had a large quantity of it prepared in pieces of various sizes, expressly for the use of dentists, and that it may be had of Mr. Leach of this city, and at the establishments of Messrs. Stockton & Co., in Philadelphia and New York. For sharpening excavators and every other description of dental instruments, it is by far superior to any thing we have ever used. While it cuts away an instrument with great rapidity, it at the same time leaves a perfectly smooth and keen edge upon it. We have several pieces that were presented to us by Dr. Vancamp, of Nashville, one of the owners of the quarry, which we use for smoothing the surface of plugs in teeth, preparatory to applying the burnisher, and we have found them ex ceedingly valuable for this purpose. We would advise every dentist, who would have the satisfaction of operating with sharp instruments, to procure one or more pieces of this valuable stone; and while upon the subject we would suggest to Dr. V. the propriety of furnishing his eastern agencies with an abundant supply of pieces shaped like the pinion file of a clock, for smoothing the surfaces of plugs in the sides of teeth.—*Balt. Ed.*

